# Layer-by-Layer Cell Encapsulation for Drug Delivery: The History, Technique Basis, and Applications

**DOI:** 10.3390/pharmaceutics14020297

**Published:** 2022-01-27

**Authors:** Wenyan Li, Xuejiao Lei, Hua Feng, Bingyun Li, Jiming Kong, Malcolm Xing

**Affiliations:** 1 Department of Neurosurgery, First Affiliated Hospital, Army Medical University, 30 Gaotanyan Street, Chongqing 400038, China; lwy243@tmmu.edu.cn (W.L.); xuejiaolei@tmmu.edu.cn (X.L.); fenghua@tmmu.edu.cn (H.F.); 2 Department of Orthopaedics, School of Medicine, West Virginia University, Morgantown, WV 26506, USA; bili@hsc.wvu.edu; 3 Department of Human Anatomy and Cell Science, University of Manitoba, 745 Bannatyne Avenue, Winnipeg, MB R3E 0J9, Canada; 4 Department of Mechanical Engineering, University of Manitoba, 75 Chancellors Circle, Winnipeg, MB R3T 5V6, Canada

**Keywords:** layer-by-layer, cell encapsulation, cell nanocoating, cell surface engineering, drug delivery

## Abstract

The encapsulation of cells with various polyelectrolytes through layer-by-layer (LbL) has become a popular strategy in cellular function engineering. The technique sprang up in 1990s and obtained tremendous advances in multi-functionalized encapsulation of cells in recent years. This review comprehensively summarized the basis and applications in drug delivery by means of LbL cell encapsulation. To begin with, the concept and brief history of LbL and LbL cell encapsulation were introduced. Next, diverse types of materials, including naturally extracted and chemically synthesized, were exhibited, followed by a complicated basis of LbL assembly, such as interactions within multilayers, charge distribution, and films morphology. Furthermore, the review focused on the protective effects against adverse factors, and bioactive payloads incorporation could be realized via LbL cell encapsulation. Additionally, the payload delivery from cell encapsulation system could be adjusted by environment, redox, biological processes, and functional linkers to release payloads in controlled manners. In short, drug delivery via LbL cell encapsulation, which takes advantage of both cell grafts and drug activities, will be of great importance in basic research of cell science and biotherapy for various diseases.

## 1. Introduction

Recent decades have witnessed the rapid development of cell encapsulation. Cell encapsulation, which is a form of cell surface modification, leads to material film coatings on cell membranes. This technique has drawn much attention in various fields, such as cell therapy, cell biosensors, biocatalysts, and so on [1,2,3]. Further, since it provides an artificial microenvironment surrounding cells, it can also be viewed as a platform for investigating interactions between cells and extracellular matrix (ECM).

Based on the cell number, it can be sorted as an encapsulation of cell mass and single cell [4]. The encapsulation of cell mass is to coat cell group with materials in order to evaluate the cellular function as a whole. On the contrary, single-cell encapsulation endows surface coating via material “shell” on individual cells. Thus, it facilitates the exploration of homogenized influences on single cells and become attractive in recent years [5]. With the progress in single-cell encapsulation, eukaryocytes, especially stem cells, are gradually attempted to serve as the core, and the technique becomes an interdisciplinary field of chemistry, biology, and material science.

Among the diverse range of methodologies for building up cell encapsulation, layer-by-layer (LbL) self-assembly is a well-defined one [6]. The strategy yields the stacking of polyelectrolytes by their opposite charges and enables the building of thin films in a fast, economical, and controllable way [7]. Since the cell membrane is negatively charged, a LbL technique is naturally applicable to establish a permeable, biocompatible, and modifiable capsule in single-cell level, exerting great potentials in various applications.

## 2. History of LbL Single-Cell Encapsulation

**Figure 1:**
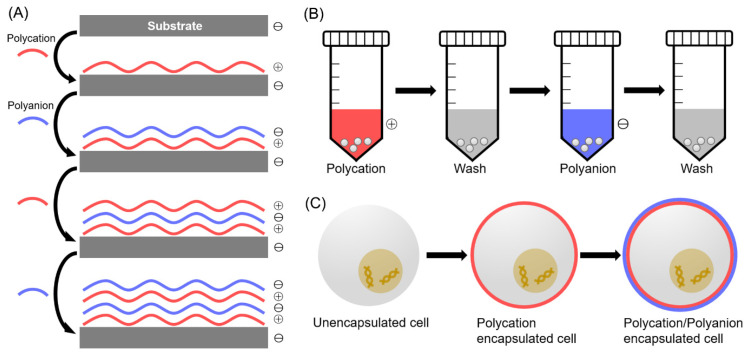
Scheme of the LbL cell encapsulation. (**A**) Negatively charged substrate is incubated with polycation and polyanion alternatively to stack polyelectrolyte films on the substrate based on the electrostatic force. (**B**) Cells in a centrifuge tube are incubated with polycation, followed by centrifuge and washing with buffer solution. Polyanion is then introduced in the same way. (**C**) Cell surface is modified with multilayers using LbL self-assembly.

The LbL self-assembly technique which leverages electrostatic adsorption was introduced by Decher in early the 1990s [8]. Since then, it has attracted significant attention in a diverse range of fields, for example, chemistry, nano-materials, and biology [9,10,11]. Among these attempts, cell encapsulation has emerged as a cutting-edge technique (Figure 1).

In 2000, Möhwald et al. firstly introduced cell encapsulation via LbL. In their research, fixed erythrocytes were encapsulated using poly(allylamine hydrochloride) (PAH) and poly(styrene sulfonate) sodium salt (PSS) [12]. Fixed *Escherichia coli (E. coli)* was also encapsulated with these polyelectrolyte pairs by Neu et al. [13]. Not long after, people began to encapsulate live cells with LbL. In 2007, Sugunan et al. utilized gold nanoparticles (AuNPs) in the encapsulation of live fungi [14]. These works have evidenced the feasibility of LbL on cells, but they generally regard cells as the encapsulating core and thus overlook the functions. Indeed, these materials are even cytotoxic for mammalian cells with complicated functions [15,16].

Researchers started to pay attention to the cellular function in encapsulation on mammalian cells. Lvov and colleagues coated poly(dimethyldiallylammonium chloride) (PDDA) and PSS on platelets. They proved that the tuning of encapsulation conditions would affect the functions of platelets [17]. Surface characteristics of encapsulated cells, such as roughness and stickiness, were then investigated in a followed study [18]. Further, Diaspro et al. found that proliferation of cells could be affected by coating with PAH and PSS [19]. These early works demonstrated that single-cell encapsulation via LbL technique can impose various influences on cells, such as viability, migration, proliferation, and differentiation, laying the foundation of future applications.

Investigations into applications of single-cell encapsulation began to arise in 2009, when erythrocytes were protected from immune attack via encapsulation which plays a role in camouflaging [20]. It was also applied to evaluate the nutrient uptake of *Bacillus subtilis* (*B. subtilis)* from ECM and the proliferation rate [21]. Since the polyelectrolytes have reactive sites, Li and Lin loaded growth factors on these materials for cell encapsulation to regulate functions of stem cells and dermal cells [22,23]. Mooney et al. even modified the sensitivity of mesenchymal stem cells to cytokines through encapsulation and grafted them in mice [24]. In 2020, researchers managed to manipulate the ingredient of cell membrane, for example glycan, via click-chemistry to form adjustable encapsulation [25]. It is worth noting that some materials or strategies are inappropriate for mammalian cells due to the effect of cell membrane destruction by some polycations [26]. Therefore, for probing the applications of single-cell encapsulation, proper choice of materials is important.

## 3. Biomaterials Employed in LbL Cell Encapsulation

Materials used in LbL encapsulation can determine the success of single-cell coating to a large extent. Polycations with positive charges have been described as possessing characteristics such as anti-peroxidation, anti-tumor, and anti-inflammation [27,28]. Polyanions with negative charges are broadly applied in drug delivery, chemical synthesis, and so on [29,30]. Chain structure, electrostatic force, reactive amine groups, active chemical bonds, and nucleophilicity underpin the essential differences between different polyelectrolytes. Based on the existing work, some of these polyelectrolytes are largely used in coating of non-mammalian cells, and some are mainly used in mammalian cells (Table 1). The difference is greatly determined by cytotoxicity and application.

### 3.1. Polycations Utilized for Non-Mammalian Cell Coating

Polycations can either come from natural extraction or chemical synthesis. Some polycations, especially synthetic ones with strong electro-positivity, are usually applied in encapsulation of non-mammalian cells that are protected by their cell wall. Synthetic materials are manufactured in standard processes, so that the functional groups on them are highly consistent. Therefore, the application using synthetic polycations tends to become predictable and controllable [31].

#### 3.1.1. Polyethylenimine (PEI)

PEI is by far the mostly used polycationic materials in LbL. It can be synthesized to form a linear or branched structure. By hydrolysis of poly(2-ethyl-2-oxazoline), solid linear PEI can be obtained. For the viscous branched form, polymerization of aziridine is required. Since PEI contains large amount of reactive amino groups, it can be conveniently functionalized. For example, branched PEI was proved to bind DNA to constitute co-polymers for enhancing transfection rate [32].

However, the toxicity and non-degradability of PEI have apparently impeded its applications in biomedicine [33]. Amino modification, for instance, disulfide bond crosslinking and acylation [34,35], has been performed to lessen the cytotoxicity. Apart from these complicated procedures, modification of the primary amines with trimethylene carbonate is suggested to be an easy approach [36]. As to the improvement of degradability, researchers found that addition of cleavable bonds to PEI chains could sufficiently aid its breakdown, for example, ester conjugation [37].

**Figure 2:**
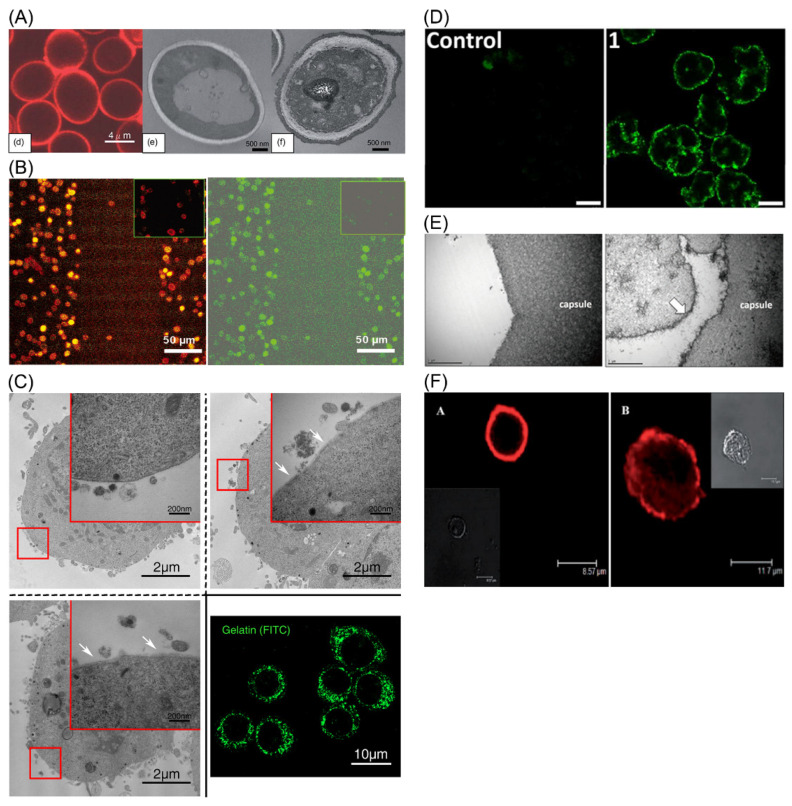
LbL cell encapsulation with polyelectrolytes. (**A**) Yeast cells encapsulated with PEI, tannic acid and poly(N-vinylpyrrolidone) were verified with fluorescence labeling and TEM. Adapted with permission [38]. Copyright 2011, The Royal Society of Chemistry. (**B**) Fluorescence imaging of cells encapsulated with PAH and PSS. Adapted with permission [40]. Copyright 2005, American Chemical Society. (**C**) PC12 cells were encapsulated with gelatin and HA, and underwent TEM and fluorescence imaging. Adapted with permission [48]. Copyright 2018, American Chemical Society. (**D**) Rat islets were coated with polyamidoamine and alginate. Adapted with permission [63]. Copyright 2013, American Chemical Society. (**E**) TEM demonstrated the encapsulation by chitosan and alginate. Adapted with permission [76]. Copyright 2021, Korean Endocrine Society. (**F**) MSCs encapsulated with PLL and HA were investigated with fluorescence and brightfield imaging on different time points. Adapted with permission [87]. Copyright 2007, John Wiley and Sons.

Since PEI is such a potent polycation, it can only be used in non-mammalian cell encapsulation. Yeast cells were coated as the core with PEI as the protecting layer by Kozlovskaya et al. It was demonstrated that encapsulation films could be maintained for six days (Figure 2A). This multilayered shell was permeable to nutrients so that cellular functions remained intact [38].

#### 3.1.2. PAH

According to literature on the subject, poly(allylamine phosphate) can react with hydrochloric acid to give rise to product of PAH. It is hydrophilic since there are a large number of hydrogen bonds within its structure [39]. PAH is popular in cell encapsulation owing to its solubility and relatively weaker electro-positivity. Krol et al. encapsulated yeast cells with PAH in 2005, illustrating the effects of molecule filtration and sheer force resistance (Figure 2B) [40]. Some even incorporated nanotubes with PAH to encapsulate yeasts for stabilizing the coating capsules [41].

#### 3.1.3. PDDA

PDDA is produced by polymerization of diallyldimethylammonium chloride to gain varied molecular mass and the procedure requires peroxide as the catalyst [42]. The high density of positive charges on PDDA enables it to be suitable in cell coating. Researchers encapsulated *Allochromatium vinosum* with PDDA and PSS as the polyelectrolyte pair, demonstrating the metabolism of cells still remained unaffected [43]. Similarly to other synthetic polycations such as PEI, PDDA is not appropriate for surface engineering for mammalian cells because of its cytotoxicity.

### 3.2. Polycations Utilized for Mammalian Cell Coating

As it is well acknowledged, the lack of the protection by cell wall makes mammalian cells more vulnerable than non-mammalian cells in most cases. Therefore, materials applied on cell membrane require additional attentions especially for selection of polycations. Generally, there are only natural polycations and a few synthetic ones being verified as adequate candidates in mammalian cell coating since they are degradable, biocompatible and low-immunogenic [44,45]. Furthermore, chemical modification of the materials is still feasible due to the reactive sites, suggesting the various successful applications [5]. Naturally, these materials are usually applicable for both mammalian and non-mammalian cells.

#### 3.2.1. Gelatin

Gelatin is extracted from the natural source collagen. Owing to its biocompatibility, low-immunogenicity, and degradability, gelatin has been broadly applied in biomedicine. Its structure complex consists of 18 amine groups, with lysine and arginine showing positive charges. Gelatin has two subtypes, which are determined by manufacturing methods. Acidic degradation gives rise to type A with the isoelectric point (IEP) from 6.0 to 9.0; basic degradation generates type B with the IEP of 4.7 to 5.4. It is believed that asparagine and glutamine are degraded into carboxyl groups during the reaction, decreasing the IEP of type B gelatin. The Food and Drug Administration (FDA) of the United States has evidenced the safety of gelatin in food industry, which guaranteed its extensive use in fields of material and medicine [46,47]. In most cases, type A gelatin receives more attention since it becomes cationic in a neutral environment, and is able to combine with negatively charged surfaces, which includes the cell membrane. For example, encapsulation of cell line and neural stem cells (NSCs) with gelatin were realized, showing no obvious toxicity (Figure 2C) [22,48].

In some instances, those that require stronger polycation, cationization of gelatin can be performed. A passive way is to reduce the pH of the medium, resulting in the protonation of amino groups on gelatin. A more common approach is to conjugate ethylenediamine or spermine to gelatin under the catalyzation of 1-ethyl-3-(-3-dimethylaminopropyl) carbodiimide hydrochloride (EDC). As a result, gelatin becomes more cationic and is used for various applications [49,50].

#### 3.2.2. Cationic Cellulose

This natural polycation consists of a linear conformation by β-1, 4-D-glucans. It has been verified to be degradable, hydrophilic, and anti-microbial, therefore cellulose and the derivatives are ideal for therapeutic uses [51].

Cationic cellulose is obtained through the etherification of alkylene epoxides and glycidyl ammonium; however, the yield of stable product is challenging. The explanation is due to the poor solubility of cellulose which results from the plentiful hydrogen bonds [52]. Kaldeus et al. managed to esterify methacrylate co-polymers with cellulose composite to be amphiphilic and the mechanical features were greatly improved [53].

In addition to esterification, cellulose can react with hydroxyethyl and hydroxypropyl. The former procedure requires substitution of polyethylene glycol to produce polyquaternium derivatives for enhancing drug delivery [54]. Hydroxypropyl cellulose is synthesized by addition of poly (2-dimethylamino ethylmethacrylate) to form a comb-like structure. It has already been approved by FDA because of its biocompatibility [55].

However, the effect of encapsulation using cellulose is controversial. Singh et al. encapsulated *Lactobacillus* with cellulose and demonstrated satisfied viability of encapsulated cells [56]. Insulin-secreting cells which are acknowledged as INS1E were shown to be encapsulated with cellulose, and the formed artificial islet-like structure exhibited intact cell survival and sensitivity to glucose [57]. However, in an early study, inflammation was found to be provoked by transplanted cells encapsulated by cellulose [58]. Therefore, basic characteristics of cellulose should be deeply clarified for future application of cellulose encapsulation.

#### 3.2.3. Polyamidoamine

As a synthetic polycation, polyamidoamine demonstrates marked features, such as biocompatibility and biodegradability. It is obtained by conjugating bis-acrylamides on the backbone via Michael addition. Since the sidechains of backbone are able to be modified, polyamidoamine is regarded to be versatile.

Traditionally, linear polyamidoamine has been proved to be a competent DNA carrier [59]. To improve the cargo delivery and release, the active sites arranged on backbone can be modified with acetal or ketal for hydrolyzation when pH decreases [60]. Sometimes, hydrolyzed oligoamines can be functionalized with disulfide bonds via Michael reaction. The active disulfide bonds would endow polyamidoamine with better biocompatibility and higher efficiency in gene transfection [61,62].

In addition to DNA delivery, polyamidoamine is well-known in encapsulation of mammalian cells. The phosphine-conjugated polyamidoamine exhibits attenuated toxicity and, thus, the coating on islets does not interfere cell functions. Furthermore, the encapsulation structure demonstrates protective effects for islets against immune responses (Figure 2D) [63].

#### 3.2.4. Chitosan

Chitosan is extracted from shells of crustaceans. There are N-acetylglucosamine and D-glucosamine in its constitution with varied ratios, length, and arrangement. It also has a pKa of 6.0 to 6.5, which is approximately weak acidic under neutral environment, implying potentials in biomedicine studies [64].

When pH is adjusted to lower than 6.0, chitosan gains higher density of charges to be cationic with increased toxicity. However, when the environmental pH becomes neutral, solubility of chitosan is lowered to generate suspending aggregates. Some people tried to adjust acetylation and molecular mass to alter the electric charges. Owing to its natural source and cationic charges, chitosan is broadly applied to bind with anionic substances, including the cell membrane [65]. Nonetheless, when applied in biomedicine, the low solubility is a remarkable flaw since neutral pH is required in most occasions. Some demonstrated that chitosan swollen distinctly in aqueous condition to cause burst release of carried drugs [66].

Fortunately, chitosan can be chemically modified to cope with these difficulties. It has been verified that each chitosan unit consists of one reactive amino group and two reactive hydroxyl groups. By means of amino alkalinization and side chain modification, features such as amine protection can be introduced without the impact on the initial characteristics, because the process is not affecting the pH [67]. In addition, solubility of chitosan in solutions with a wide range of pH value can either be enhanced. Further, viscidity is suggested to be in direct proportion with the extent of alkalinization, benefiting the incorporation of chitosan with other molecules. Specifically, the direct way of alkalinization is to react methyl iodide with chitosan under alkaline environment, with trimethyl chitosan chloride as the most frequently applied form [68,69]. Additionally, there are other methods of chitosan modification. For example, quaternary ammonium of chitosan with the feature of bacteria resistance was fabricated by Jia et al. [70]. Chitosan was also coupled with folate in varied ratios under catalyzation of EDC for delivering chemotherapeutic agents [71]. Alternatively, glycidyl-trimethyl-ammonium chloride was crosslinked with chitosan to generate N-((2-Hydroxy-3-trimethylammonium) propyl) chitosan chloride which presented ideal solubility and permeability under neutral condition in the presence of the tight junctions within the molecules [72]. Meanwhile, conjugation of poly-L-lysine (PLL) will benefit chitosan with higher positive charges, reduced toxicity and enhanced transfection rates even when compared with PEI [73].

In cell encapsulation, chitosan has been widely used in multiple cell types including mesenchymal stem cells (MSCs) [74,75]. In spite of its mechanical instability, chitosan was explored to encapsulate islets together with alginate as grafts in diabetes animals (Figure 2E) [76]. Zhu et al. improved the stability in cell encapsulation by applying N-acetylated chitosan, but cytotoxicity increased accordingly [77]. In short, selection of chitosan derivatives needs special carefulness.

#### 3.2.5. Poly-L-lysine (PLL)

PLL which consists of vast amount of primary amino groups is a commonly used synthetic polycation. Via electrostatic force, PLL can be easily coupled with negatively charged substances post protonation. In synthesis, each primary amine protects one lysine monomer, converting into cyclic anhydride which then undergoes a ring-open process to yield PLL. As such, constitution of PLL can be altered simply by tuning ratios of the initiator and lysine [78].

In LbL technique, PLL has been popular in establishing polyelectrolyte films. With a pH value of 7.0, primary amines of PLL can undergo partial protonation and render PLL the ability to buffer from pH 5.7 to 7.7. It is notable that modification of molecular weight by adjusting amine groups is of huge influence since the PLL with a high molecular weight exhibits significant cytotoxicity, and that with a lower one showed alleviated toxicity but increased instability [79,80].

To deal with these obstacles, people produced branched functionalized derivatives. For instance, enhanced ability of buffering and decreased deposition can be realized by PEG addition. Additionally, bioactive regulators, such as the artery wall binding peptide, were demonstrated to be introduced to PEG-PLL, through the crosslinking of cysteine [81]. Moreover, drug delivery in a tissue-targeting manner is applicable with chemically modified PLL. Lactic acid incorporation on Doxorubicin-loading PLL was proved to assist the targeting to liver tissue. Through the interaction of JL1, leukemia cells were proved to be targeted [82,83,84].

To reduce the toxicity of PLL, its dendrimers are usually applied. Initially, by crosslinking with lysine and benzhydrylamine, researchers obtained intermediate product to be bidirectional asymmetric. The lysine derivative can be activated via acid-triggered deprotonation and then generate PLL dendrimer [85]. Afterwards, fabrication of dendrimers is largely processed under alkaline conditions. For example, researchers managed to obtain PLL derivatives with reduced toxicity as well as higher chargers via coupling of arginine, succinimyldipropyldiamine and lysine under alkaline environment [86].

At the same time, PLL has been broadly used in LbL coating for mammalian cells. It was applied with hyaluronic acid (HA) in the LbL encapsulation of MSCs. These materials form a multilayered shell with a thickness of 6.0 to 9.0 nm, exhibiting no adverse effects on cell morphology and survival (Figure 2F) [87]. In another study, Wilson and colleagues linked PEG on PLL backbone to attenuate toxicity. It demonstrates that the PEG-conjugated polymer could be used in islets encapsulation without interference of normal functions. In the animal model of diabetes, PEG-PLL-coated islets were proved to be favorable after transplantation [88].

### 3.3. Polyanions Utilized for Mammalian Cell Coating

Generally speaking, both natural and synthetic polyanions are less toxic. Therefore, researchers have a wide selection in performing LbL technique.

#### 3.3.1. Alginate

Alginate is a natural polyanion from algae. This polysaccharide has a linear structure with variedly arranged residues of α-L-guluronic acid and 1, 4′-linked β-D-mannuronic acid. The constitution of alginate structure depends on the ratio of residues. Guluronic acid is of high affinity to cations, yielding the alginate with a high content of guluronic acid to be solid. Contrarily, alginate becomes soft when there is more mannuronic acid [89]. Owing to this variable feature, alginate receives great attention in material science.

Bivalent ions are sometimes used in crosslinking alginate for encapsulating capsule generating. Ca^2+^ and Ba^2+^ are more applied than Cu^2+^ and Pb^2+^ which show distinct toxicity. Specifically, with the increase in affinity of ions, rigidity of alginate capsules was tremendously raised [90].

As indicated, pores are easily formed in the alginate capsules, thus still facilitating immune recognition and thereafter reactions [91]. Researchers proved that Ba^2+^-alginate could only exempt allogeneic grafts from immune attack, instead of the xenogeneic ones [92]. To overcome the obstacle, some researchers applied polycations to bind with alginate, such as PLL and poly-L-ornithine [93,94]. Additionally, PEG, glutaraldehyde and cellulose can also play the similar role [95,96,97].

Although pores on alginate shell can be covered by LbL coating with polycations, it is important to control the pore size to allow transportation of necessary nutrients and normal functions of surface molecules. Kendall et al. demonstrated that following LbL coating with PLL/poly-L-ornithine and alginate, sensitivity of islets to blood glucose was hindered. The extent of impact was largely dependent on the thickness of the multilayers instead of the islet properties. To be specific, the negative influence of encapsulation could be significantly eliminated when material concentration was less than 0.1% and coating time within 10 min. Otherwise, the permeability of encapsulation was reduced to give rise to disordered metabolism [98].

Rigid encapsulation with alginate can also be performed via covalent crosslink. Some applied photoactive crosslink for establishing alginate encapsulation, but the process led to obvious toxicity since plenty of redox was generated [99]. Incorporation of hydroxyl and aldehyde groups into alginate is another way to stabilize encapsulation, as well as retain permeability [100,101].

Interestingly, the purity of alginate in encapsulation can also influence the immunoreaction. It is because proteins and endotoxins contained in alginate may induce intensive immune attack when they diffuse [102]. Additionally, it is assumed pathogen-associated molecular pattern molecules with a conserved structure exist in alginate that is impure, activating immune responses and eliciting inflammation [103]. Li and colleagues established encapsulation of NSCs with gelatin and alginate, showing satisfied biocompatibility and neglectable inflammation [22].

#### 3.3.2. HA

HA utilized in biomedicine and material science is often obtained from connective tissues. Molecular structure of HA is strictly conserved, consisting of 1,4-b-N-acetyl-D-glucosamine and 1,3-b-D-glucuronic acid. It broadly exists in almost all organism, especially in skin and joint since HA is able to preserve moisture. Meanwhile, the HA which supports ECM plays an important role in the interaction among cells. It also exhibits biodegradability by hepatic cells or hyaluronidase in plasma [104].

An in vivo study showed that HA in blood circulation was immobilized on glycosaminoglycans and membrane receptors via electrostatic interaction. Notably, receptors of HA are not unique and contain several types, such as CD44, intercellular adhesion molecule-1, and hyaluronan mediated motility receptor. Since CD44 is verified to be expressed by tumor cells, HA-CD44 interaction provides a tumor-targeting strategy in design of drug delivery. In short, due to its satisfied features such as bioaffinity, biodegradability and tumor-targeting, HA has been extensively reputed in chemistry, pharmacy, and bioengineering [105].

HA can undergo chemical modifications since it has reactive sites, such as N-acetyl, hydroxyl and carboxyl groups. Hydroxyl groups can be simply modified through esterification etherification, or divinyl sulfone coupling. Carboxyl groups modification by amidation or esterification is usually mediated by carbodiimide [106]. Partial and terminal modification generate two common subtypes of HA derivatives [107]. Generally speaking, the partially modified HA is able to be covalently conjugated so that it meets the demands in various applications. At the same time, it should be noted that toxicity, accompanied with the chemical modification, maintains biocompatibility. On the contrary, since terminally modified HA cannot form covalent bonds, its application in bioengineering is limited. Other functional modifications of HA were also reported. For example, the RGD motif was combined with HA to enhance the adhesion [108]. Some even applied methacrylic anhydride to endow HA with photoreactivity [109].

Owing to the natural affinity with ECM, HA attracts great attention in cell engineering, including LbL encapsulation. It has been suggested that MSCs can be encapsulated with HA in chondrogenesis enhancement and supporting matrix fabrication for cartilage regeneration [110]. HA was also utilized in cell encapsulation for nervous system diseases to promote neural repair and glia cell activation [111].

#### 3.3.3. PSS

PSS is a synthetic polymer with advantageous hydrophilicity and thermoplasticity. It has already been used to encapsulate red blood cells and islets [112]. However, the weak mechanical rigidity hinders its application, even though PSS and PAH pair prevents immunoreaction when used in LbL encapsulation [113]. Åkerfeldt et al. demonstrated that encapsulation structure with PAH and PSS was easily destructed by shear force [114]. Even worse, the complement system activation was triggered by PSS, leading to impaired survival of encapsulated mammalian cells [115].

### 3.4. Other Materials

**Figure 3:**
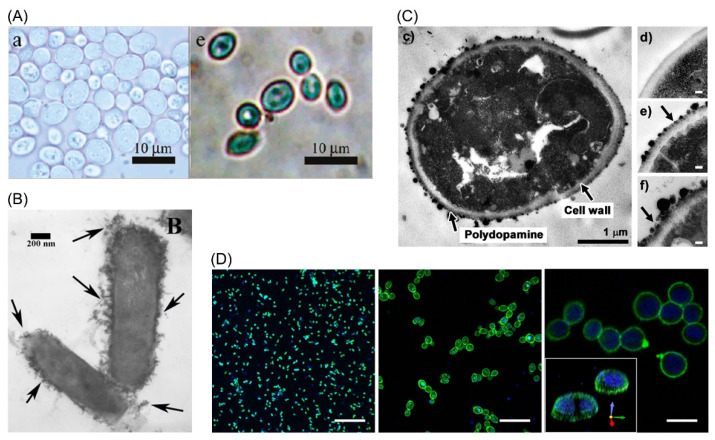
LbL cell encapsulation with atypical methodologies. (**A**) Optical imaging of uncoated yeasts and yeasts coated with Ag nanoparticles. Adapted with permission [116]. Copyright 2009, American Chemical Society. (**B**) *Borkumensis* was encapsulated with magnetic nanoparticles as exhibited under TEM. Adapted with permission [119]. Copyright 2016, American Chemical Society. (**C**) TEM showed mussel-inspired encapsulation of yeast cells. Adapted with permission [124]. Copyright 2011, American Chemical Society. (**D**) Bacteria, eukaryotic cells and mammalian cells were proved to be encapsulated with fluorescence-labeled DNA polymers. Adapted with permission [126]. Copyright 2019, The Authors.

In addition to polyelectrolytes, which are natural or synthetic, nanoparticles can be utilized in single-cell coating. Au and Ag are commonly used particles which were applied to encapsulate fungi. Surprisingly, the coated fungi exhibited contrary phenomena in biological processes and morphologic features (Figure 3A) [116]. To improve the electrochemical features, such as conductivity, carbon nanotubes were used to encapsulate *S. cerevisiae* [117]. Graphite oxide also presented increased conductivity and antibacterial activity with biological compatibility [118].

Meanwhile, magnetic nanoparticles have drawn attention in fabrication of bioreactors, biosensors, and single-cell encapsulation since they provide the encapsulation system manipulability through magnetic field (Figure 3B) [119]. In a study, encapsulation of yeast cells with magnetic nanoparticles still maintained the enzymatic activities, so that a manipulable biocatalyst was produced [120]. Since the nanoparticles directly contact cells, they influenced the cell survival unavoidably. To deal with the problem, PAH was linked to magnetic nanoparticles and improved viability was achieved owing to the prevention of particles from entering cells [121]. This modified nanoparticle was further applied in multi-types of mammalian cells to explore the cellular functions since the viability of mammalian cells could be preserved [122]. It should be noted that most nanoparticles would be internalized into cells as time passed, and the extent was related to the properties of cell membrane which affected interactions. Cells in rapid proliferation tended to internalize nanoparticles faster [123].

Biology-inspired materials are also attractive due to their bioaffinity. Mussel-inspired LbL coating takes advantage of well-defined adhesiveness of mussel which is attributed to polydopamine. Yang et al. coated polydopamine on yeast cells individually and found that cell survival could be preserved and proliferation manipulated (Figure 3C) [124]. Another study applied dopamine and HA to construct LbL films. This scaffold was shown to be effective in eliminate reactive oxygen species (ROS) and promote osteogenesis, and thus demonstrated great promises in cell therapy [125]. Deoxyribonucleic acid (DNA) is another bio-derived polymer in LbL coating. It was used in encapsulation of multiple cell types and provided a precisely programmed encapsulation pattern (Figure 3D) [126].

**Table 1:** Representative examples of materials applied in LbL cell encapsulation.

Materials		Cell Types	Applications	Reference
Polycations (for non-mammalian cells)	PEI	Yeast cells	Function maintenance	[38]
	PAH	Yeast cells	Cell immobilization	[40]
	PDDA	*Allochromatium vinosum*	Physical protection	[43]
Polycations (for mammalian cells)	Gelatin	PC12 cells; NSCs	Protection and payload delivery	[48,49]
	Cationic cellulose	Islet cells	Function maintenance	[57]
	Polyamidoamine	Islet cells	Immunocamouflage	[63]
	Chitosan	MSCs; Islet cells	Directed differentiation; Fibration prevention	[75,76]
	PLL	MSCs	Function maintenance	[87]
Polyanions	Alginate	Fibroblasts, endothelial cells	Oxidative stress reaction control	[101]
	HA	MSCs; NSCs	Directed differentiation	[110,111]
	PSS	Islet cells	Immunocamouflage	[115]
Others	Metallic nanoparticles	*S. cerevisiae*	Function maintenance	[116]
	Carbon nanotubes	*S. cerevisiae*	Toxicity evaluation	[117]
	Graphite oxide	*S. cerevisiae*	Antimicrobial	[118]
	Magnetic nanoparticles	*Alcanivorax borkumensis;* Yeast cells; HeLa cells	Cell functionalization; Spatial locomotion	[119,120,122]
	Mussel-inspired polydopamine	Yeast cells	Cell protection	[124]
	DNA molecules	*Escherichia coli,* Yeast cells, MCF-7 cells	Function maintenance	[126]

## 4. Interactions of LbL Multilayers with Interfaces

LbL self-assembly is based on electrical adsorption which will change the properties of attached substances. Again, the interaction itself also affects the multilayers features. In this context, a comprehensive understanding of principles of LbL multilayers is important for further applications.

Till now, people have obtained detailed understanding of the characteristics of interfaces. To be specific, the interacting interfaces can be classified into planar surface and colloidal particle. Techniques, such as surface forces apparatus, atomic force microscope, and total internal reflection fluorescence, have come into use for investigation of interacting forces with the planar surface [127,128,129]. On the other hand, colloidal particles are studied via rheological measurement, time-resolved light scattering, and turbidity measurement [130,131,132]. Strong polyelectrolytes can always have stable charges, such as branched PEI and PSS. Conversely, the electrical property of weak polyelectrolytes is greatly influenced by environmental pH and ions, such as type A gelatin, linear PEI, and PLL. By titration and electronic field measurement, extent of ionization was evaluated [133].

### 4.1. Properties of Materials Adsorption

The interaction of polymers with the attached substances is based on the electrostatic force. Due to the fact that most polyelectrolytes are hydrophobic, interactions are largely dependent on force of van der Waals and hydration [134].

#### 4.1.1. Interactions on Planar Interfaces

Observation of materials stacking was conducted by Kargl et al. [135]. It was found at the beginning, the thickness of multilayers increased, indicating that polyelectrolytes binding was in progress. With time going on, encapsulation thickness did not increase due to the saturation of surface materials [136]. When the surface was rinsed, LbL multilayers were still maintained, indicating that the interaction within LbL self-assembly was firm [135]. However, detachment might happen to small-molecule polymers and it was particularly obvious when solution ingredients varied [137].

As suggested, the degree of material attaching is proportional to concentration, which was even verified in a math model [127]. This effect is common in most cases, although hydrodynamics affects it sometimes [138]. As concentration of polyelectrolytes increased, the adsorption speed increased accordingly. They explained it with limited relaxation of the multilayers since the lateral relaxation of encapsulating polymers would be hindered by fast attachment of the surrounding materials when concentration increased. This mechanism generally existed in studies of protein interaction [139].

#### 4.1.2. Interactions on Colloidal Particles

Polyelectrolytes are able to be absorbed on colloidal surfaces once LbL self-assembly is performed. Similar to situations for planar surface, materials can attach to particles to reach saturation when excessive materials exist. Therefore, polyelectrolytes should be more widespread for a homogenous distribution on particles, otherwise there would be discrepancy in degree of encapsulation on the surface [140]. The formed multilayers were proved to be irreversible [141]. Additionally, through electrophoresis, the amount of materials on surface of particle can be precisely obtained. Nonetheless, the dynamic kinetics in the encapsulation process could still influence the results more or less [142].

### 4.2. Properties of Polyelectrolyte Multilayers

#### 4.2.1. Amount of Adsorbed Materials

In encapsulation with PDDA and SiO_2_ in aqueous solution of 50 mM salt, the saturated mass of PDDA was approximately 0.3 μg/mm^2^ since one layer of adsorbed atom was 1.0 to 2.0 μg/mm^2^. Further, when polyelectrolytes interaction reached saturation, the adsorption became 0.01 to 1.0 μg/mm^2^ [143].

The adsorbed mass of materials is closely related to materials’ innate characteristics and solution ingredients. Molecular weight of polyelectrolytes, concentration of ions and electrical charge density are key factors. To be specific, the amount of branched polyelectrolytes in LbL adsorption is proportional to the molecular mass [144]. Meanwhile, the importance of polymer concentration was suggested by Godman et al. They found that interaction of polyelectrolytes could be reversed under the circumstances of weakly charged polyelectrolytes or high-concentration solutions [145].

There is great variation in polyelectrolytes’ charge density which influences hydrophility accordingly. By means of adding weak ions or tuning environmental pH, polymers’ charge density may be significantly affected. It should also be noticed that the influence is different between bound and unbound LbL multilayers, since ionization may be in progress with LbL assembling. In particular, the adsorbed materials increased with high charges of substrates and low charges of materials, and absorption reaches to maximum with the polymer charge at a low level [146].

#### 4.2.2. Morphology of Polyelectrolyte Multilayers

In LbL self-assembly, polymers attach to each other by electrostatic adsorption. The chains generate repelling force at the same time and thus lead to the formation of potential space within multilayers. Meanwhile, the electrostatic force which is attractive within multilayers flattens chains of polyelectrolytes, leading to heterogeneously arranged thin films. On the contrary, the polymer films are homogeneous when weak polyelectrolytes and solution of strong ions are applied [133].

Moreover, Xu and colleagues applied techniques, such as dynamic light scattering and quartz crystal microbalance in measuring thickness of the multilayers, which was suggested to be at nanometer-scale [147]. Based on the fact that polymer chains diameter was from 20 to 100 nm, it indicated that these chains were significantly compressed during the LbL self-assembly procedure. Implied from the layer thickness, water accounted for around 20–60% in the pressed materials. Yet, in solutions of high-concentration salt, multilayers tended to swell and form pores. Selin et al. proved that LbL films were thickened when salt concentration and molecular weight of materials were elevated, as well as the charge was reduced [148].

Although a similar conclusion can be drawn via dynamic light scattering, the result obtained from surface techniques is larger and indicates the swollen material films. Although the difference may be determined by different substrates, it is also explained by sub-layers existing in a single film. The thickness of the sub-layer is greatly dependent on the solution concentration, giving rise to the observed differences by the two methodologies. It is noteworthy that systematic errors in these techniques cannot be fully avoided since the LbL layers are ultra-thin and easily influenced by environment [149].

Lateral heterogeneity of attached materials has been verified by atomic force microscopy. It is extremely significant when there are potent polyelectrolytes and a low salt solution. With the LbL self-assembly using branched PAA, researchers verified that even if multilayers interaction reaches saturation, potential space is still within the films because the branched materials possess repulsive force [150]. The extent of lateral heterogeneity of branched PAMAM films was also quantified with atomic force microscopy at molecule-scale [151]. For films of linear materials, although their statistical features can be obtained via atomic force microscopy, detailed delineation of saturated multilayers of attached linear materials is still lacking [152]. In the meantime, it was acknowledged that PAMAM-PSS films were heterogeneous, examined by direct force measurement [153]. Therefore, the above studies suggested that linear polyelectrolytes are inclined to form LbL multilayers with homogeneity. In addition, similar to the disturbed lamellar phase, homogeneous multilayers are usually formed by hydrophobic polyelectrolytes which are weakly charged [154].

### 4.3. Charge Balance of LbL Multilayers

#### 4.3.1. Charge Reversal

Charge reversal is a pivotal characteristic of LbL adsorption, resulting from overcharging. It is well known that polyelectrolytes and substrates bind because of electrostatic force. Therefore, coating materials may fall from the substrates if they possess the same charges. Nonetheless, since adsorbed polyelectrolytes in LbL self-assembly are heterogeneous, only the features of substance in assembly are crucial. In this context, once there is one empty binding site, a molecule of polyelectrolyte can attach [155]. In addition, there are also interacting forces within the LbL formation, such as van der Waals and hydrophobic forces, which are unaffected by interface charges [156,157].

Detection of polymer charges can be performed with electrokinetic techniques which involve electrophoresis and surface potential monitoring. The former method is for measuring electrophoretic kinetics of particles via mathematic models. The latter one can be used for detection of planar substrates by relative values obtained through direct force measurement [158]. Specifically, amidine particles were applied to exhibit the charge reversal for particles [159]. As it was proved, charges of non-coated particle are reversed by adsorbed polymers to show opposite charges. During the LbL self-assembly, when polyelectrolyte is added to reach the threshold, surface charge of particles becomes neutralized. With the continuous addition of polyelectrolyte to be excessive, charges on particles accumulate until saturation [140]. Measurement of charge reversal on planar substrates was conducted via streaming potential detection on poly (allyl amine)-mica interaction [160]. With the increased amount of polyelectrolyte, saturation was gradually reached and the charges of surface were reversed. They suggested an incubation time of 20 min and application of low-concentration materials to attenuate the reaching of plateau.

#### 4.3.2. Charges Distribution in the Multilayer Structure

Patterns of charge density and surface potential have already been described. For example, when the surface is positively charged, these local charge groups are compensated by the reduction in positive charges and accumulation of negative charges. The cationic charges of saturated polyanion are overcompensated to give rise to anionic surface [161]. The slow progress of the compensation implied the decreased net charges on the substrate.

The details of variation in charge distribution within LbL structure can be examined. The mechanism is based on the threshold value of charge reversal, since chemical titration is used to inspect the charges when the interface charge becomes neutral on this threshold. Additionally, counterions play an important role in charge balance, especially for dendritic materials and low-charge substrates. For instance, counterions are responsible for the compensation of 90% of charges generated in LbL films by PEI and PAA. Even for saturated films, only a very little part of the charges is compensated by free polyelectrolytes. The lateral deviation led by heterogeneous polyelectrolytes can likely explain the differences residing in adsorbed materials and exhibited charges [162].

The charge reversal point shift can be interpreted with the solid stoichiometry which demonstrates the necessary demand for weakly charged polymers in the charge compensation [163]. Similar to this tendency, a small amount of polyelectrolytes is required for compensating the low charges on substrates. Simultaneously, the requirement for pH of solutions which involve weakly charged polymers can be naturally acknowledged [164]. The reduction in pH may lead to an increase in the charge of weak polycations, therefore the reversal threshold inclines to shift to higher pH with strengthened polyelectrolyte interaction on surface with solid charge distribution [165]. This same situation happens in the combination of weak-acid or amphiprotic substrates with strongly-charged cationic materials. Under the contrary conditions that feeble polyanions adsorb to cationic substrate or potent polyanion attaches to feeble alkaline or amphiprotic surfaces [166]. However, the findings could not be applied to pH-sensitive materials and surfaces.

As verified, the shift of reversal point is also related to changes of molecular weight. For instance, when molecular weight of materials increases, the charges are greatly compensated in a PAA adsorption study. Therefore, the net charge increase lags behind molecular weight increase, and, thus, the reversal point deviates to high material doses. However, it is not applicable for linear polymers because they often interact to form a more flattened pattern, and the variations in stoichiometry are not proportional to molecular mass changes [167].

## 5. Functional Regulation of Cells by LbL Encapsulation

In recent years, regulation of cell functions, such as viability, proliferation, differentiation, migration, and immune disguise, has attracted significant attention. Yang and Wang have illustrated that the encapsulation shell prevented cells from biological, chemical, and physical damages [168,169]. In addition to protection, functional regulation can be realized by modification of materials. It is certain that an appropriate duration of encapsulation polymers is required for regulating cellular functions.

**Figure 4:**
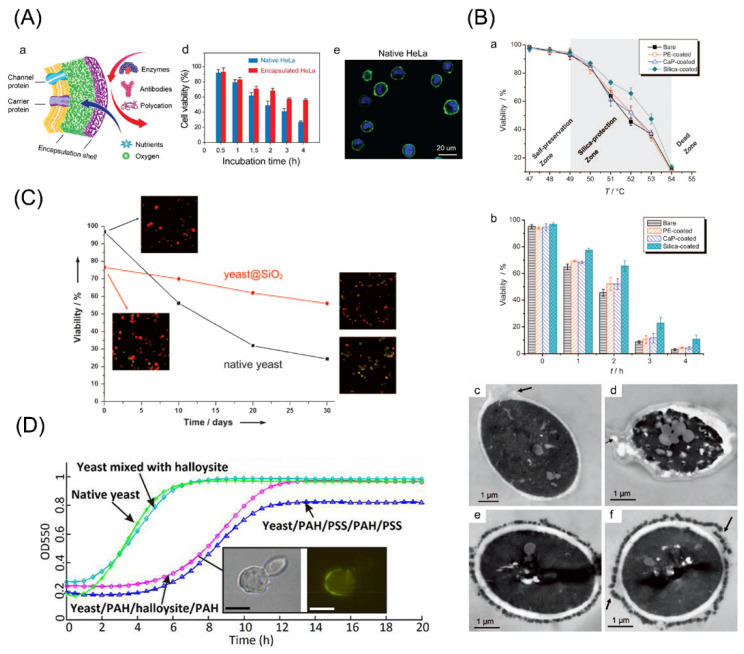
Functional regulation by cell encapsulation. (**A**) Yeasts can be protected from enzymes by encapsulation with gelatin and PEG. Adapted with permission [171]. Copyright 2017, Elsevier. (**B**) Thermo-resistance imposed by cell encapsulation. Adapted with permission [173]. Copyright 2010, John Wiley and Sons. (**C**) Encapsulated cells exhibited higher viability in water for a long term. Adapted with permission [174]. Copyright 2009, John Wiley and Sons. (**D**) Proliferation of yeast cells can be tuned by encapsulation. Adapted with permission [41]. Copyright 2013, Royal Society of Chemistry.

Biological hazards can be protected from functioning. Nguyen et al. encapsulated yeasts with a PAH and PSS pairing, and found that the cells were protected from enzymatic activity of lysosomes [170]. Moreover, mammalian HeLa cells which were encapsulated with gelatin and PEG could be greatly protected from the lytic enzymes, such as trypsin. In the latter case, more than 50% of coated cells stayed alive, and there were only 27% of untreated cells surviving [171] (Figure 4A). The mechanism was regarded as the reduced permeability of the adverse factors by encapsulation.

Protection of encapsulation against high temperature has also been verified. González-Ferrero et al. kept encapsulated *Lactobacillus* cells under heat stress and their survival could still be retained [172]. The heat-resistance effect was also investigated in encapsulation of yeasts with SiO_2_ and it could be interpreted by its effect on water retention via the silica layers [173] (Figure 4B).

Studies have demonstrated the protective effects of encapsulation against physical factors, such as centrifuge and osmotic pressure. *S. cerevisiae* was encapsulated with silica-based multilayers and exhibited satisfied viability in water for a long period [174] (Figure 4C). Maheshwari et al. even applied nanoparticle-modified graphene in *S. cerevisiae* encapsulation to preserve them in water for one week [175]. For mammalian cells, a gelatin-fibronectin pair was used in single-cell encapsulation of HepG2. Following centrifuge with ultra-high speed, uncoated cells exhibited a survival rate of 10% while the encapsulated cells show the rate of over 80% [176].

Sometimes encapsulation may negatively influence the cellular functions. Yeast proliferation was shown to be interfered following encapsulation with SiO_2_ multilayers and the effect could be reversed when thinner SiO_2_ layers were formed [41] (Figure 4D). The reason may be underlay by the obstruction of nutrients and wastes. Further, mechanical properties of the encapsulation multilayers, for example rigidity, were suggested to pose a great impact on the proliferation of coated cells. Ishihara and colleagues performed encapsulation on HeLa cells, demonstrating that the increased rigidity of encapsulation structure could obviously inhibit the proliferation; and the proliferation rate could be recovered when coating layers were disrupted [177].

## 6. Applications of Drug Delivery via LbL Cell Encapsulation

The controllable drug delivery has great potential in many diseases with a deteriorative progression. These diseases usually require sustained effects which can hardly be obtained from traditional therapies. Nanoparticles, such as lipid-based carriers, were usually applied in drug delivery for these diseases, since they exhibited a large surface area for drug loading and different binding sites for hydrophilic/hydrophobic/amphiphilic molecules [178]. However, the cytotoxicity, tissue irritation, and uncontrolled diffusion were supposed to be the drawbacks [179,180]. In comparison, LbL cell encapsulation often applied natural polymers which avoided cell or tissue damage after transplantation, and was able to protect encapsulated cells from damaging factors via molecule isolation and permit diffusion of nutrients for metabolism [181]. Additionally, the incorporation of payloads on encapsulation structures helped the controlled release of drugs. Therefore, the “cell encapsulation + payloads” complex was expected to facilitate the simultaneous transplantation of cells and bioactive agents, showing great potentials in transplantation therapy.

**Figure 5:**
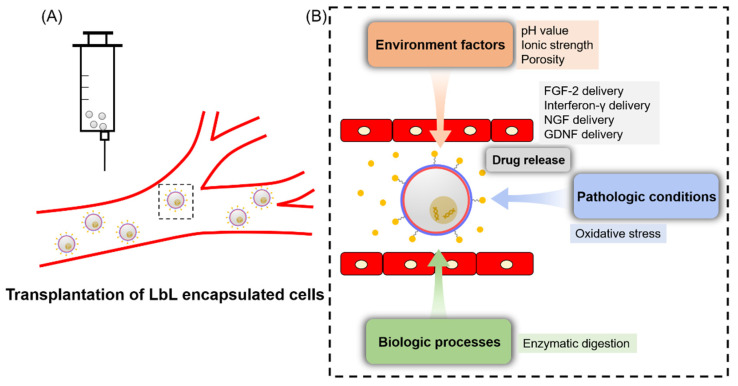
LbL cell encapsulation for drug delivery can be tuned by multiple factors. (**A**) This technique can be applied in cell transplantation therapy for various diseases. (**B**) Drug delivery is able to be manipulated by different factors and representative works are listed.

Payload diffusing from the LbL multilayers and the degradation of LbL materials largely determine the speed and degree of drug release from the LbL structure [182]. The multilayers degradation was verified to be resulted from material degradation and interaction of ions with counterions and film swollen [183,184]. Controlled drug delivery via LbL benefited due to reduced cytotoxicity, lowered drug dose and enhanced therapeutic effects (Figure 5).

To achieve a sustained drug release from LbL cell encapsulation, material layers can be thickened and covalent conjugation of drugs can be applied [185,186]. On one hand, due to the multilayers structure, staged drug releasing of multiple regulators can be realized in previous studies [187,188]. On the other hand, a triggered release is required under some circumstances. Therefore, responsive materials are employed in the formation of LbL encapsulation [189]. The triggerable feature can be the innate property of coating materials, such as the pH-sensitivity in weakly charged polymers, or crosslinking of responsive groups on materials [190,191]. There are also strategies of LbL encapsulation in cell targeting by materials’ nature or functional modification. For example, CD44 which is predominant on tumor cells can be specifically recognized by HA [192]; conjugation of antibodies on polyelectrolytes facilitates the targeting of LbL structure to the ligands of interest [193]. As such, desired materials on demand are able to be constructed for aiming at specialized therapeutic outcomes by LbL cell encapsulation.

### 6.1. Environment-Adjustable Release

By utilization of polyelectrolytes in LbL encapsulation, especially the weak ones, the electrostatic interaction can be conveniently adjusted by different stimuli [154]. Yuan and co-workers synthesized porous materials for LbL drug delivery, and the process can be tuned by pH. To be specific, when pH value was higher than 9.6, a sustained release over 16 days was achieved; when lower than 3.2, a large number of residual drugs were kept in materials for at least 3 months [194]. The feature is attractive in anti-cancer research since the pH in the tumor microenvironment is acidic. For example, Zhao and colleagues developed a doxorubicin delivery system via encapsulation with chitosan and HA, showing a faster releasing rate in carcinogenic sites than in others [195]. Additionally, co-delivery of drugs via LbL in a pH-triggered manner has been extensively applied by Li’s group these years, and it brought the on-demand delivery and sustained release of multiple drug types [196,197,198].

Some people adjusted ionic strength to control the LbL architecture and, thus, the loaded compounds. For LbL multilayers with PDADMAC and PAA, when the ionic strength was elevated to 0.6 M, disassembly of LbL films took place [199]. Similarly, hydrogen bond-based encapsulation could also be tuned by the addition of salt solutions [200].

Porosity of LbL encapsulation can become another factor. In a study applying PAH and PSS as the encapsulation polyelectrolytes, an increase in ethanol proportion in the medium greatly affected the porosity of multiplayers and then facilitated the payload release [201]. The controlled delivery of urease was realized by Lvov et al. by taking advantage of this feature [202]. Although this is effective in modifying drug delivery, the addition of ethanol can inhibit the bioactivity of the carried compounds to some extent.

### 6.2. ROS-Controlled Release

Due to the fact that peroxidative conditions exist in many different diseases, ROS-responsive materials have received great chances for inducing controlled payload release following transplantation [203]. Presumably, the ROS-responsive encapsulation structure keeps relatively stable in circulation, and it is disrupted to release drugs after getting into the pathologic sites which present the condition of abundant ROS. Yan and co-workers fabricated the ROS-responsive encapsulation system in poly(vinylpyrrolidone) and poly(methacrylic acid) multilayers which were coupled with bisazide as the triggering linker for conveying DOX [204]. This controlled delivery system exhibited higher efficiency in cancer cell elimination. In another case, researchers applied folic acid as the ROS-sensitive linker in the encapsulation architecture to deliver Luteolin, demonstrating ROS-triggered delivery and anti-tumor effects [205].

### 6.3. Biology Induced Release

A remarkable feature of this strategy is the independence on external inducers. Physiological factors and enzymes can be two major types in the biology-controlled drug delivery. The former factors involve pH value and salt components, which can probably influence the cargo release from LbL multilayers without external stimuli [206]. Under the physiological conditions, compounds release is a passive process after the multilayers gradually disassemble or results from the interruption of chemical bonds between compounds and the polyelectrolytes [207,208]. The latter methodology utilizing enzymatic activity is based on materials which are also the substrates of enzymes. This has been proven in LbL encapsulation with polysaccharides and peptides. For example, LbL multilayers formed by poly(lactic acid) were degraded via the effect of α-chymotrypsin [209]. In animal studies, proteases were demonstrated to digest LbL films constructed with dextran and poly(L-arginine). Another study took advantage of an enzyme which was put inside LbL films with poly(L-lactide) and poly(D-lactide) to adjust their degradation time [210].

### 6.4. Drug Delivery via Cell Encapsulation

In many diseases, cell transplantation presents great potential for providing cell supplementation and nutrient secretion. As previously mentioned, LbL encapsulation protects grafted cells and those diverse strategies of drug delivery via LbL self-assembly further aid the effect of cell therapies.

Fibroblast growth factor-2 (FGF-2) was incorporated on gelatin and modified gelatin, and could form an LbL structure with alginate on dermal papilla cells, which are stem cells residing in hair follicles. The growth factor was suggested to be conveyed to aid hair cell survival and proliferation. The strategy was also verified to be effective in subcutaneous transplantation [23]. Ham and colleagues crosslinked interferon-γ on coating materials and then performed LbL encapsulation on neural stem cells. The crosslinked compound successfully promoted the neuronal differentiation of stem cells. Furthermore, in vivo experiments proved the improvement in development of neural tube by the interferon-γ-modified encapsulation of neural stem cells [211].

In cell therapy for nervous system diseases, the blood–brain barrier is always an obstacle for delivering therapeutic compounds into brain tissue. The drugs should also maintain a certain quantity and quality to generate effects. LbL cell encapsulation as a carrier is bringing increasing prospects in treating central nervous system disorders such as Huntington’s Disease [212,213]. Nerve growth factor (NGF) was tethered with polyethersulfone in the fibroblast encapsulation for treating neurodegenerative diseases [214]. Researchers took advantage of human retinal epithelial cells, which are known as ARPE-19 in the cell encapsulation. Astonishingly, these coated cells retained their viability in the brain and eye for years, since the well-defined coating films protected cells from the inflammatory factors and the grow factor effects simultaneously [215,216]. The reinforced glial cell-derived neurotrophic factor that was released from C2C12 myoblasts was designed by Perez-Bouza et al. and the transplantation of which provided a sufficient source of the neurotrophin for Parkinson’s Disease patients [217].

## 7. Conclusions

This review summarized the basics and applications of LbL encapsulation. Different types of materials, both natural and synthetic, can be utilized in the formation of multilayers. This technique views cells not just as the physically encapsulated cores, but as functionally modified organisms. The details of interaction within LbL films were also sophisticatedly depicted. Since LbL encapsulation works as a capsule on cells, it certainly affects the cellular functions. Even more, the coating materials can be modified to gain the required functions in drug delivery under certain conditions, for example, targeting delivery, sustained delivery, multi-load delivery, and responsive delivery. These features largely extended the applications of LbL cell encapsulation in either basic or clinical researches.

It is a certainty that LbL cell encapsulation is still in its infant stage. There are challenges involving design of responsive materials and delicate regulation of cellular function under specific disease environment. The shortage of clinical trials is another concern to further apply this engineering strategy. Nonetheless, in the future, LbL cell encapsulation will contribute to the development of diverse fields in creative ways.
